# TNNI3K, a Cardiac-Specific Kinase, Promotes Physiological Cardiac Hypertrophy in Transgenic Mice

**DOI:** 10.1371/journal.pone.0058570

**Published:** 2013-03-05

**Authors:** Xiaojian Wang, Jizheng Wang, Ming Su, Changxin Wang, Jingzhou Chen, Hu Wang, Lei Song, Yubao Zou, Lianfeng Zhang, Youyi Zhang, Rutai Hui

**Affiliations:** 1 Sino-German Laboratory for Molecular Medicine, State Key Laboratory of Cardiovascular Disease, FuWai Hospital & Cardiovascular Institute, Chinese Academy of Medical Sciences, Peking Union Medical College, Beijing, People's Republic of China; 2 Department of Cardiology, State Key Laboratory of Cardiovascular Disease, FuWai Hospital & Cardiovascular Institute, Chinese Academy of Medical Sciences, Peking Union Medical College, Beijing, People's Republic of China; 3 Key Laboratory of Human Disease Comparative Medicine, Ministry of Health, Institute of Laboratory Animal Science, Chinese Academy of Medical Sciences and Comparative Medical Center, Peking Union Medical College, Beijing, People's Republic of China; 4 Institute of Vascular Medicine, Peking University Third Hospital, Beijing, People's Republic of China; University of Illinois at Chicago, United States of America

## Abstract

**Purpose:**

Protein kinase plays an essential role in controlling cardiac growth and hypertrophic remodeling. The cardiac troponin I-interacting kinase (TNNI3K), a novel cardiac specific kinase, is associated with cardiomyocyte hypertrophy. However, the precise function of TNNI3K in regulating cardiac remodeling has remained controversial.

**Methods and Results:**

In a rat model of cardiac hypertrophy generated by transverse aortic constriction, myocardial TNNI3K expression was significantly increased by 1.62 folds (P<0.05) after constriction for 15 days. To investigate the role of TNNI3K in cardiac hypertrophy, we generated transgenic mouse lines with overexpression of human TNNI3K specifically in the heart. At the age of 3 months, the high-copy-number TNNI3K transgenic mice demonstrated a phenotype of concentric hypertrophy with increased heart weight normalized to body weight (1.31 fold, P<0.01). Echocardiography and non-invasive hemodynamic assessments showed enhanced cardiac function. No necrosis or myocyte disarray was observed in the heart of TNNI3K transgenic mice. This concentric hypertrophy maintained up to 12 months of age without cardiac dysfunction. The phospho amino acid analysis revealed that TNNI3K is a protein-tyrosine kinase. The yeast two-hybrid screen and co-immunoprecipitation assay identified cTnI as a target for TNNI3K. Moreover, TNNI3K overexpression induced cTnI phosphorylation at Ser22/Ser23 *in vivo* and *in vitro*, suggesting that TNNI3K is a novel upstream regulator for cTnI phosphorylation.

**Conclusion:**

TNNI3K promotes a concentric hypertrophy with enhancement of cardiac function via regulating the phosphorylation of cTnI. TNNI3K could be a potential therapeutic target for preventing from heart failure.

## Introduction

In response to increased workload, the heart undergoes hypertrophic enlargement, which is characterized by an increase in the size of individual cardiac myocyte.[1] This hypertrophic response can be traditionally classified as either physiological or pathological. Physiological stimuli such as exercise lead to compensatory growth of the cardiomyocyte, accompanied by normal cardiac structure, preserved or improved cardiac function, and minimal alteration in cardiac gene expression pattern.[2] In contrast, the pathological hypertrophy, which is induced by persistent pressure or volume overload at various disease conditions, is associated with reactivation of fetal gene program, interstitial fibrosis, cardiac dysfunction and eventual heart failure.[3] As heart failure is almost invariably associated with cardiac hypertrophy, the elucidation of signaling cascades involved in these two forms of hypertrophy will be of critical importance for the design of specific therapy against heart failure.[4], [5]

Protein kinase plays an essential role in regulating cardiac growth and hypertrophic response. Various kinases transmit hypertrophic signals from membrane bound receptors and change the phosphorylation status of functionally significant proteins.[6] Cardiac myofilament, the ultimate determinant in the control of cardiac contractility, is a central feature of kinase signal transduction. Levels of contractile protein phosphorylation are associated with stretch of the myocardium, the myofilament response to Ca^2+^ and the progression of cardiac remodeling.[7]–[9] Despite considerable progress has been made in elucidating the roles of various kinases in regulating myofilament during the past decades, understanding the molecular mechanism underlying myofilament phosphorylation and cardiac hypertrophy remains limited. This is due to, at least in part, lack of knowledge for the function of novel protein kinases in the heart. In this regard, it is crucial to identify novel genes potential involved in cardiac hypertrophy.

The cardiac troponin I-interacting kinase (TNNI3K), also known as CARK, is a novel cardiac-specific kinase. It contains a central kinase domain, flanking by an ankyrin repeat domain in the amino terminus and a serine-rich domain in the carboxyl terminus. TNNI3K is a functional kinase and directly interacts with cardiac troponin I (cTnI). [10] It has been suggested as a factor that moderates electrocardiographic parameters and the susceptibility for viral myocarditis. [11], [12] Our group has verified that Mef2c, an important determinant for cardiac hypertrophy, play a critical role in regulating basal TNNI3K transcription activity.[13] The precise function of TNNI3K in regulating cardiac remodeling, however, has remained elusive and controversial. Some studies have shown that TNNI3K induces cardiomyocyte hypertrophy *in vitro* [14] and enhances cardiac performance and protects the myocardium from ischemic injury *in vivo*, [15] while others have shown that overexpression of TNNI3K can accelerate disease progression in mouse models of heart failure. [16]

To better understand the role of TNNI3K in cardiac remodeling, we generated transgenic mice overexpressing TNNI3K specifically in the heart. Our data demonstrated that increasing basal TNNI3K expression resulted in enhancement of cardiac function and adaptive hypertrophy. Furthermore, TNNI3K directly interacts with cTnI and induced cTnI phosphorylation at Ser22/Ser23 *in vivo* and *in vitro*. These data suggest that TNNI3K promotes cardiac remodeling via regulating the phosphorylation of cTnI.

## Materials and Methods

### 2.1. Ethics Statement

All animal experiments were approved by the FuWai Administrative Panel for Laboratory Animal Care and were consistent with the Guide for the Care and Use of Laboratory animals published by the United States National Institutes of Health. Rats and Mice were housed in an AAALAC-accredited facility with a 12-hour light-dark cycle and allowed water and food ad libitum. All surgery was performed under anesthesia, and all efforts were made to minimize suffering.

### 2.2. Transverse aortic constriction (TAC) surgery

Male Sprague-Dawley rats (200–250 g) were anesthetized with 2% isoflurane. Adequacy of anesthesia was assessed by monitoring the respiratory rate as well as the loss of response to toe pinch. The rats were then intubated and ventilated using a rodent ventilator (Model 683, Harvard Apparatus, South Natick, MA, USA). Midline sternotomy was performed, and the transverse aorta was exposed. The aorta was ligated between the innominate and left common carotid arteries by tying a 7–0 silk suture around a tapered 22-gauge needle placed on top of the aorta. Sham-operated controls underwent an identical surgical procedure including isolation of the aorta, only without placement of the suture. At different time points (day 1 to day 15) after surgery, animals (n = 4 to 5 for each time point) were euthanized by overdose anesthesia (pentobarbital sodium 150 mg/kg, i.p.) and cervical dislocation. The hearts were removed; left ventricles were weighed and quickly frozen in liquid nitrogen for total RNA extraction.

### 2.3. Quantitative real-time PCR analysis

Total RNA was isolated from left ventricular tissue using Trizol and reverse transcribed with Superscript III transcript kit (Invitrogen, Carlsbad, CA, USA). SYBR green-based quantitative real-time PCR was carried out with the DNA Engine Opticon 2 real-time PCR Detector (BIO-RAD, Richmond, CA, USA) as previous described.[13] Melting curve analysis was used to confirm amplification specificity. GAPDH gene was used as internal control. The primers are listed in Table S1. All experiments were repeated at least twice in triplicate.

### 2.4. Plasmid Constructs and Generation of Transgenic Mice

A human wild-type TNNI3K cDNA (2508 bp, NM_015978) was subcloned into the *SalI/HindIII* site of between the 5.5-kb murine α-myosin heavy chain promoter (α−MHC) and the 0.6-kb human growth hormone (hGH) polyadenylation sequence, carried in the pBluescriptII-SK+ vector (Stratagene). The transgenic mice were generated in the key laboratory of Human Disease Comparative Medicines as previously described[17]. Briefly, an 8.7-kb DNA fragment was isolated, purified from transgenic vector after digestion with *NotI*, and microinjected into fertilized oocytes from C57BL/6J mice. The surviving eggs were surgically transferred into pseudopregnant females. The resulting pups were screened by diagnostic PCR using the primers for *TNNI3K*, 5′-ATG GCA AGA GCA TTG ACC TAG TC-3′ and 5′-GGA TGA TTG AGC TGG CAG AGA-3′. The *Fabpi* gene was amplified as internal control using the primers, 5′-TGG ACA GGA CTG GAC CTC TGC TTT CCT AGA-3′ and 5′-TAG AGC TTT GCC ACA TCA CAG GTC ATT CAG-3′. To determine the transgene copy number, Southern blot analysis was performed on tail genomic DNA digested with *EcoRI* and probed with a ^32^P-labeled 0.6 kb *HindIII/NotI hGH* fragment. The purified transgene insert DNA was added into the *EcoRI* digested wild-type mouse DNA to yield the equivalent of 1, 5, and 10 copies of the gene per haploid genome (based on 3×10^9^ base pairs per haploid genome). The signals were quantified using ImageJ, and the copy number was determined from the standard curve. Three independent founder lines were identified and mated to C57BL/6J wild-type mice. Transgenic hemizygous mice were born, studied, and compared with their wild-type counterparts.

### 2.5. Northern Blot Analysis

Transgenic mice and their wild-type counterparts were sacrificed by cervical dislocation at the age of 3 months. Total RNAs were isolated using Trizol (Invitrogen, Carlsbad, CA, USA) from multiple organs, including heart, liver, spleen, lung, and kidney. Aliquots (20 µg) of total RNA were separated on 1% agarose gels containing 2.2 M formaldehyde and were blotted on Hybond N+ membrane (Amersham Pharmacia, Piscataway, NJ, USA). The probe was a 611 bp TNNI3K cDNA fragment amplified from the transgenic vector using the following primers, 5′-AAA GAT TAG AAG ATG ACC TGC-3′ and 5′-ATC TTG AGC ATT CAC ATC TG-3′. The probe was labeled with ^32^P using a Random Primer DNA Labeling Kit (TaKaRa, Dalian, China) based on supplier's protocol. After hybridization, the membranes were washed, and exposed to films (Kodak). The signal was detected using ImageJ software.

### 2.6. Echocardiography

Mice were weighted and anesthetized with 2.5% avertin (0.018 mL/g) given i.p. Adequacy of anesthesia was monitored by lack of reflex response to toe pinch. Two-dimensional short- and long-axis views of the left ventricule (LV) were obtained by transthoracic echocardiography with the Vevo 770 Imaging System and a 30-MHz probe (VisualSonics, Toronto, Canada). M-mode tracings were recorded and used to determine LV end-diastolic diameter (LVEDD), LV end-systolic diameter (LVESD), and LV posterior wall thickness (LVPWT) and interventricular septum (IVS) in diastole over three cardiac cycles. LV fractional shortening (FS) was calculated with the formula %FS = (LVEDD−LVESD)/LVEDD. After echocardiography examination, the mice were sacrificed by cervical dislocation. The hearts were excised, rinsed in ice-cold saline, weighed, dissected into left and right ventricles, frozen in liquid nitrogen and stored at −80 °C.

### 2.7. In Vivo Hemodynamics Analysis in Transgenic Mouse

Non-invasive hemodynamic analysis was performed in 3-month-old TNNI3K transgenic mice and age-matched littermate controls as previous described.[18] Mice were anesthetized with an intraperitoneal injection of 2.5% avertin (0.018 mL/g). Adequacy of anesthesia was monitored by lack of reflex response to toe pinch. A 1.4 French Millar catheter-tip micromanometer catheter (SPR-719, Millar Instruments Inc, Houston, Texas) was inserted through the right carotid artery into the left ventricular. After stabilization for 10 min, the pressure signal was continuously recorded on a computer. The peak LV systolic pressure and LV end-diastolic pressure were measured, and the maximal slopes of systolic pressure increment (dP/dt_max_) and diastolic pressure decrement (dP/dt_min_), indexes of contractility and relaxation, respectively, were analyzed.

### 2.8. Histological and Morphometric Analysis

Hearts from transgenic mice and nontransgenic littermate controls were collected and fixed in 4% paraformaldehyde buffered with PBS, routinely dehydrated, and paraffin embedded. Hearts were sectioned at 4 µm and stained with hematoxylin and eosin, and Masson's Trichrome. Mean myocyte size was calculated by measuring 150 cells from sections stained with hematoxylin and eosin.

### 2.9. Cell Culture and Recombinant Adenovirus

Adenovirus encoding full-length human TNNI3K (Ad-TNNI3K) was constructed using AdEasy Adenoviral Vector System. The 1- to 2-day-old neonates were sacrificed by cervical dislocation and the primary ventricular cardiomyocyte was isolated by enzyme digestion as we previously described.[19] The cardiomyocytes were planted onto 35-mm-diameter wells (six well plates) at a density of ≈2×10^5^ cells per square centimeter and cultured in DMEM supplemented with 10% fetal bovine serum, 100 units/ml penicillin/streptomycin and 0.1 mM bromodeoxyuridine (BrDu). The following day the cells were washed in phosphate-buffer saline and cultured in serum-free DMEM, containing penicillin/streptomycin (100 units/ml). For adenoviral infection, cardiomyocytes were incubated for 2 hours with Ad-TNNI3K and Ad-GFP at an approximate multiplicity of infection of 50. Forty-eight hours following infection, >95% of the cells were GFP positive. The cells were harvested at basal level or after isoproterenol-stimulation for 10 min (10 nmol/l). The protein kinase activity was assessed by Western Blot.

### 2.10. Western Blots

Western blots were carried out on extracts from left ventricular or cultured cardiomyocytes. Proteinase inhibitor and phosphatase inhibitor cocktail (Roche, Basel, Switzerland) were added according to the supplier's protocol. Protein concentrations were determined by the method of Bradford[20] and thirty micrograms of proteins were loaded on gels. Antibodies to Akt, phospho-Thr-308-Akt, phospho-Ser-473-Akt, ERK, phospho-Thr-202/204-ERK, GAPDH were purchased from Cell Signaling Technology (Beverly, MA), Antibodies to total and phosphorylated cTnI were generous gift from Prof. Xianmin Meng. Proteins were detected by Western blotting and stained with NBT/BCIP (Promega, Madison, WI, USA) or ECL detection reagents (Amersham Pharmacia Biotech).

### 2.11. Yeast two-hybrid Screen and co-immunoprecipitation

A full-length human TNNI3K cDNA, fused to the GAL4 DNA binding domain, was used as bait in a yeast two-hybrid screen of approximately 3×10^6^ clones of a human heart cDNA library (Clontech, California, USA). The interaction domain of TNNI3K to cTnI was determined by protein truncation test using immunoprecipitation techniques. The full-length and truncated TNNI3K cDNA sequence encoding amino acids 1–421, 422–726, 727–835, 1–726, and 422–835 of TNNI3K were cloned into pCMV-Myc vector between *EcoRI* and *XhoI* sites to generate pCMV-Myc-TNNI3K, pCMV-Myc-TNNI3K-ANK, pCMV-Myc-TNNI3K-PK, pCMV-Myc-TNNI3K-SR, pCMV-Myc-TNNI3K-ANK+PK, and pCMV-Myc-TNNI3K-PK+SR, respectively. Full-length human cTnI cDNA was cloned into pCMV-HA vector between *EcoRI* and *KpnI* sites to generate pCMV-HA-cTnI. The integrity of all of the constructs was verified by sequencing.

H9C2 cells were transiently transfected with 2 µg of plasmid DNA using Lipofectamine 2000 (Invitrogen) according to the manufacturer's protocol. Double transfections were carried out with the pCMV-HA-cTnI and empty pCMV-Myc or each of the TNNI3K plasmids. Forty-eight hours after transfection, cells were washed with cold PBS and harvested. Protein to protein interaction was assayed by co-immunoprecipitation using ProFound c-Myc-Tag IP/Co-IP Kits (Pierce Biotechnology, Rockford, IL) according to the manufacturer.

### 2.12. Immunoprecipitation of TNNI3K and Phospho Amino Acid Analysis

A full-length myc-tagged human TNNI3K DNA was expressed in H9C2 cells. The myc-tagged TNNI3K protein was immunoprecipitated using ProFound Mammalian c-Myc Tag IP/Co-IP kit (Pierce, Rockford, IL) and was analyzed by SDS-PAGE followed by immunoblotting with rabbit anti-phosphoamino acid antibody, anti-phosphotyrosine antibody, anti-phosphothreonine antibody, and anti-phosphoserine antibody, respectively.

### 2.13. Statistics

All measurement data are expressed as mean±SE. The statistical significance of differences between groups was analyzed by Student's t-test. Differences were considered significant at a P-value<0.05.

## Results

### 3.1. TNNI3K was involved in Cardiac Hypertrophy

**Figure 1:**
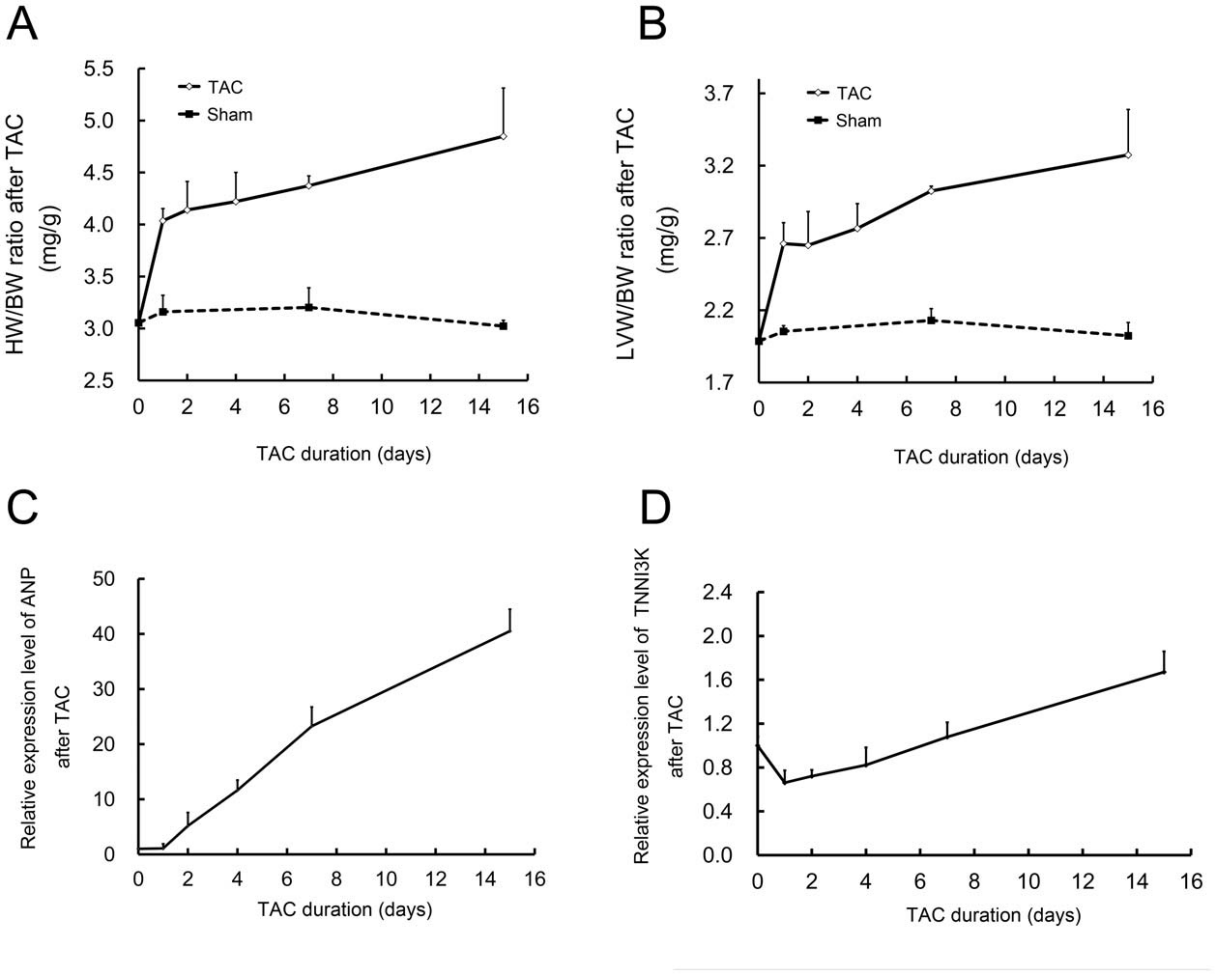
TNNI3K expression was dynamically regulated in the hypertrophic hearts in rats. A and B: the heart weight/body weight (HW/BW) and left ventricular weight/body weight (LVW/BW) were continuously increased after TAC. Five different time points were analyzed for TAC group and 3 different time points for sham operation group. Each time point included 4–5 animals. C and D: The expression of ANP and TNNI3K were detected by real-time PCR analysis. *Gapdh* was used for internal control and data were presented as fold-change compared with that of sham operation controls. Five different time points were analyzed (1, 2, 4, 7, 15 days post-operation, respectively). Each time point contained 4–5 animals.

To investigate whether TNNI3K is involved in cardiac hypertrophy, the expression of TNNI3K was examined in a rat model of cardiac hypertrophy generated by transverse aortic constriction (TAC). After constriction for 15 days, the heart weight/body weight ratio (HW/BW) and left ventricular weight/body weight ratio (LVW/BW) were increased by 59.2% and 64.3% in TAC rats compared with those of sham operated controls (both p<0.001) (Figure 1A and B). Atrial natriuretic peptide (ANP), a cardiac hypertrophic marker, was upregulated by 40 folds in TAC rats (Figure 1C). TNNI3K was significantly downregulated on day 1 (0.66 fold, P<0.05), return to basal levels on day 7, and then increased on day 15 (1.62 folds, P<0.05) (Figure 1D). This unique expression pattern indicates that TNNI3K is involved in cardiac remodeling.

### 3.2. Generation of Cardiac-Specific TNNI3K Transgenic Mice

**Figure 2:**
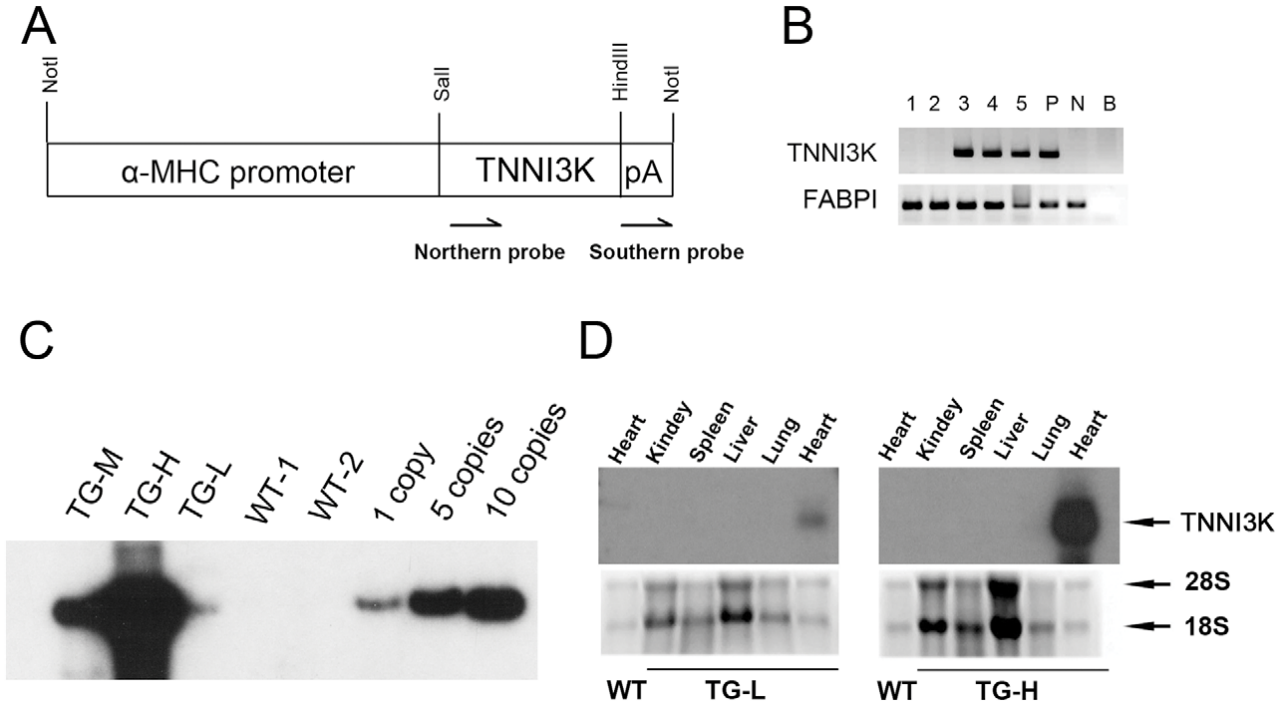
Generation of cardiac-specific *TNNI3K* transgenic mice. (A), Schematic of the TNNI3K transgene that was constructed with the α-MHC mouse promoter. pA: human growth hormone polyA sequences The positions of the Southern probe and northern probe were shown below the construct; (B), PCR genotyping of TNNI3K transgenic mice. 1–5: transgenic mice. P: positive control, wild-type mouse genomic DNA mixed with linearized transgenic fragment. N: negative control, wild-type mouse genomic DNA. B: blank, none DNA template. *FABPI* gene was amplified as internal control. (C), Southern blot analysis of wild-type and *TNNI3K* transgenic mice. Tail genomic DNA was digested with *EcoRI* and probed with *hGH* polyA sequence. Hybridization signals were present only in transgenic positive mice. Transgenic copy number was determined from the gray density against standard curve. 1 copy -10 copies: transgenic copy standards. (D). Northern blot analysis of RNA isolated from multiple tissues of the transgenic TG-L and TG-H lines. Hybridization signals were present only in the heart of transgenic mice. The RNA isolated from the heart of wild-type mouse was used as a negative control.

To investigate the role of TNNI3K as a potential regulator of cardiac hypertrophy *in vivo*, transgenic mice overexpressing the human TNNI3K specifically in heart using the mouse αMHC promoter were generated (Figure 2A). By PCR genotyping, three independent transgenic founders were identified from 33 F0 mice (Figure 2B). These founders carried 2, 8, and 44 copies of transgene, and were designated as TG-L (low copy number), TG-M (medium copy number), and TG-H (high copy number), respectively (Figure 2C). The expression of the transgene was restricted to the heart. No expression was detected in the kidneys, spleen, liver and lung (Fig. 2D). Furthermore, the transgene expression was positively associated with the transgene copy number. TG-H displayed 20-fold higher transgene expression level than TG-L (Figure 2D).

### 3.3. Overexpression of TNNI3K Induced Cardiac Hypertrophy *in vivo*

In all three lines of transgenic mice, no premature death or sign of heart failure were found after 1 year of observation. Both the TG-L and TG-M lines had no demonstrable cardiac phenotype when compared with their respective littermate controls (Table 1). In contrast, TG-H mice showed a unique profile of increased HW/BW ratio of 31.3% and 43.1% at 3 months and 12 months of age, respectively (P<0.01 and P<0.05).Therefore, further characterization studies of the transgenic phenotype were carried out using male mice from TG-H.

**Table 1:** Gravimetric Data for the TNNI3K Transgenic Mouse Heart.

	3 months				12 months	
	NTG	TG-L	TG-M	TG-H	NTG	TG-H
N	11	6	6	6	6	6
BW (g)	24.5±0.5	24.5±0.6	26.2±1.2	26.4±1.0	38.0±0.7	38.1±0.7
HW/BW(mg/g)	5.1±0.2	5.4±0.4	5.5±0.3	6.7±0.2**	5.8±0.5	8.3±0.9*
LVW/BW(mg/g)	3.6±0.4	3.5±0.4	3.6±0.2	4.4±0.3*	3.8±0.5	5.8±0.9*

Heart weight/body weight ratios and left ventricular weight/body weight ratio were calculated from TNNI3K transgenic or non-transgenic mice at the indicated time points. Values given are Mean±SE and P values were calculated by Student's t-test. *: compared with non-transgenic mice, P<0.05, **: compared with non-transgenic mice, P<0.01.

**Figure 3:**
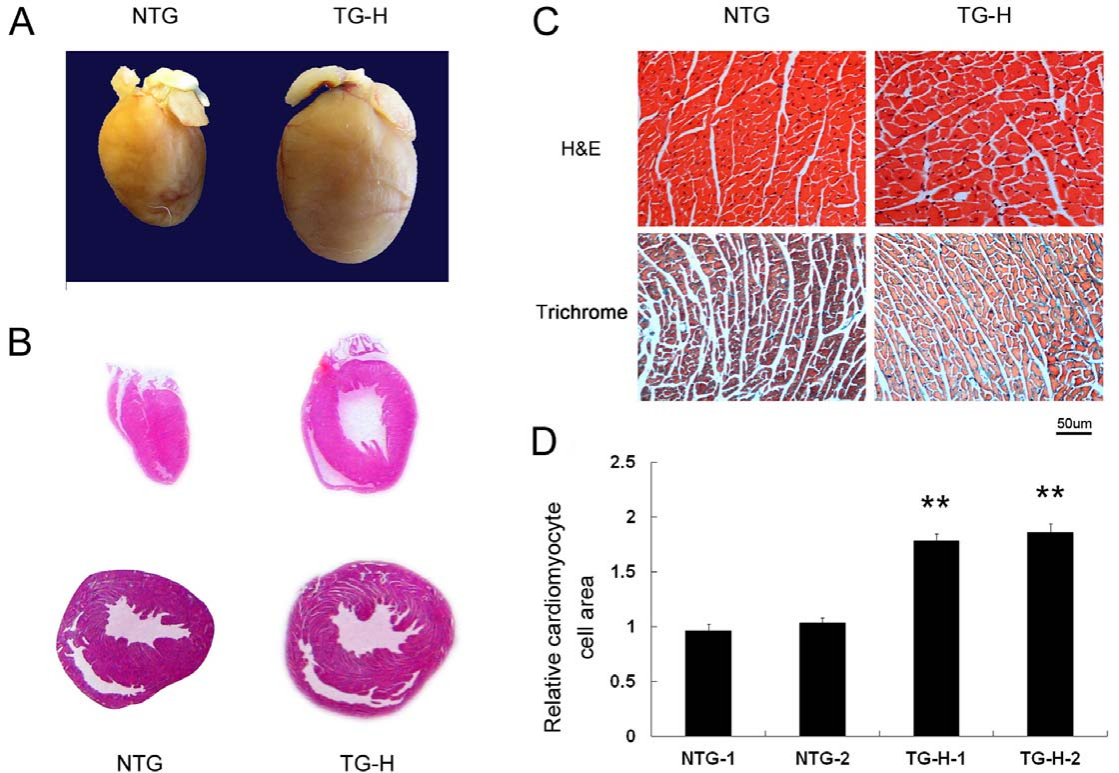
Cardiac histological analysis of TNNI3K transgenic mice at the age of 3 Months. (A) Whole heart, (B) Macroscopic view after hematoxylin–eosin-stained hearts revealed a concentric hypertrophy in TG-H mice. Upper was longitudinally sectioned. Lower was transversely sectioned. (C) Microscopic histological analysis of demonstrated cardiomyocyte hypertrophy without an increase in interstitial fibrosis in TG-H hearts. Upper panel was H&E stained sections. Lower panel was trichrome stained sections. Blue staining represents collagen deposition. Original magnification x200 (D) Cross-sectional areas of cardiomyocyte were quantified from hematoxylin–eosin-stained histological sections. At least 150 myocytes were measured each in two non-transgenic (NTG) hearts and two TG-H transgenic heart. **P<0.01 compared with wild-type.

Cardiac size was significantly larger in TG-H mice than in non-transgenic littermates at the age of 3 month (Figure 3A). Macroscopic sections showed a phenotype of concentric ventricular hypertrophy in the transgenic mice, which was characterized with smaller chamber size and thicker ventricular wall (Figure 3B). Upon microscopic observation, necrosis or myocyte disarray was not observed in TG-H mice (Figure 3C, upper panels). Masson-trichrome stain showed no interstitial fibrosis in TG-H mice (Figure 3C, lower panels). To examine whether the increase in cardiac size was due to cellular growth, the cross-section area of myocytes was quantitatively measured on the hematoxylin–eosin-stained LV myocardium of two representative transgenic mice and two controls, respectively. TG-H myocytes were substantially larger with a mean surface area 1.8-fold greater than those seen in littermate controls (Figure 3D).

To assess *in vivo* cardiac morphology and function, echocardiography were performed on TG-H and littermate control mice at the age of 3 months and 12 months, respectively. In agreement with the histological analysis, the 3-month-old TG-H mice showed a concentric cardiac hypertrophy. The left ventricular wall thickness was significantly increased (36.6% and 46.9% in IVS and LVPWT, respectively, both P<0.01), accompanied by decreased chamber dimension (19.0% and 10.8% in LVESD and LVESD, respectively, both P<0.01), and increased fraction shortening (Table 2 and Figure S1). Surprisingly, this phenotype of concentric hypertrophy persisted up to the age of 12 months (Table 2). Echocardiography showed increased ventricular wall thickness and decreased chamber dimension in the transgenic heart. More importantly, there is no loss of systolic functional performance between 3 and 12 months of age, indicating that cardiac hypertrophy in TNNI3K transgenic mice was compensated hypertrophy.

**Table 2:** M-mode echocardiograms.

	3 months		12 months	
	NTG	TG-H	NTG	TG-H
N	13	7	6	5
IVS (mm)	0.71±0.03	0.97±0.03**	0.95±0.03	1.40±0.07**
LVPWT (mm)	0.66±0.02	0.97±0.05**	0.93±0.03	1.16±0.07*
LVESD (mm)	2.32±0.06	1.88±0.10**	2.75±0.25	1.83±0.21*
LVEDD (mm)	3.79±0.05	3.38±0.14**	4.23±0.22	3.67±0.15
%FS	38.7±1.4	46.5±2.8**	35.5±3.0	50.4±4.3*

LVEDD, LV end-diastolic diameter; LVESD, LV end-systolic diameter; LVPWT, LV posterior wall thickness, IVS, interventricular septum; %FS, fraction shorting. Values given are Mean±SE and P values were calculated by Student's t-test. *: compared with non-transgenic mice, P<0.05, **: compared with non-transgenic mice, P<0.01.

To evaluate the effect of overexpressing TNNI3K on cardiac function more accurately, noninvasive hemodynamic assessment was performed using LV catheterization. After anesthesia, there was no significant difference in heart rate and blood pressure between the 3-month-old TG-H mice and non-transgenic littermates. LV pressure was mildly increased both at systolic and diastolic in transgenic heart. In consistent with the echocardiography analysis, LV dP/dt_max_ and LV dP/dt_min_ were increased by 9% and 20% in transgenic mice compared with those in wild-type mice, respectively (Table 3), indicating enhanced contractility and diastolic function for the TG-H mice.

**Table 3:** Hemodynamic Analysis of TNNI3K Transgenic Mice.

	NTG	TG-H
N	8	6
HR (beats/min)	388.5±4.8	380.5±5.7
AP systolic (mmHg)	93.0±4.6	98.0±4.2
AP diastolic (mmHg)	62.7±3.4	61.2±2.5
LVP systolic (mmHg)	104.0±3.2	125.4±11.3
LVP diastolic (mmHg)	−17.3±1.0	−13.1±1.8
LVEDP (mmHg)	−3.3±0.5	−2.2±0.3
dP/dt_max_ (mmHg/s)	6311.6±143.6	6881.8±514.6
dP/dt_min_ (mmHg/s)	−6325.3±169.6	−7567.8±720.4

HR: heart rate; AP: arterial pressure; LVP: left ventricular pressure; LVEDP: left ventricular end-diastolic pressure, dP/dt_max_ and dP/dt_min_: average maximum and minimum values, respectively, of first derivative of ventricular pressure wave.

**Figure 4:**
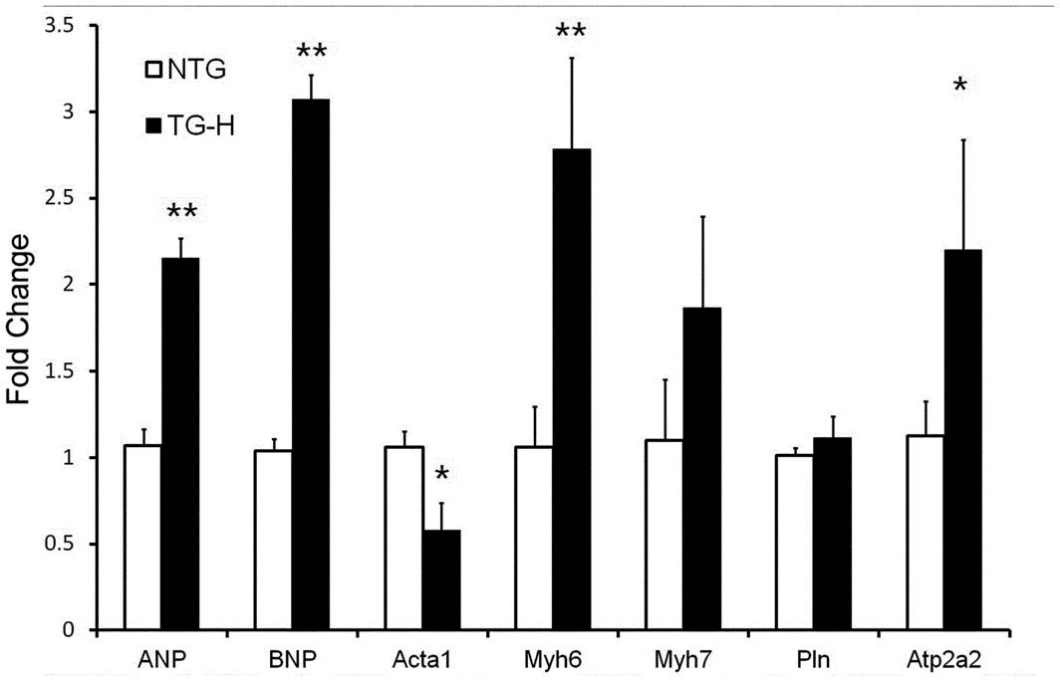
Assessment of hypertrophy marker genes. The mRNA levels were measured in left ventricle by RT-PCR analysis (N = 4 hearts in each group). *Gapdh* was used for internal control. **: P<0.01, *: P<0.05 vs nontransgenic littermates.

Cardiac hypertrophy is accompanied by reprogramming of cardiac gene expression. To characterize the molecular phenotype of TNNI3K-induced cardiac hypertrophy, we examined the transcriptional levels of a set of hypertrophic markers, including atrial natriuretic peptide (*ANP*), brain natriuretic peptide (*BNP*), skeletal muscle α-actin (*Actc1*), α- and β-myosin heavy chain (*Myh6* and *Myh7*, respectively), phospholamban (*PLN*) and sarcoplasmic reticulum Ca^2+^ ATPase (*SERCA2a*). In 3-month-old TG-H transgenic hearts, the expression of ANP and BNP was increased (P<0.01), whereas Actc1 was decreased (P<0.05). On the other hand, the expression of β-MHC was mildly increased, whereas α-MHC and SERCA2a mRNA were markedly increased (Figure 4). These discordant changes in gene expression indicate that the fetal gene program is differentially regulated in the TNNI3K transgenic heart.

### 3.4. TNNI3K does not induce the activation of ERK and Akt

**Figure 5:**
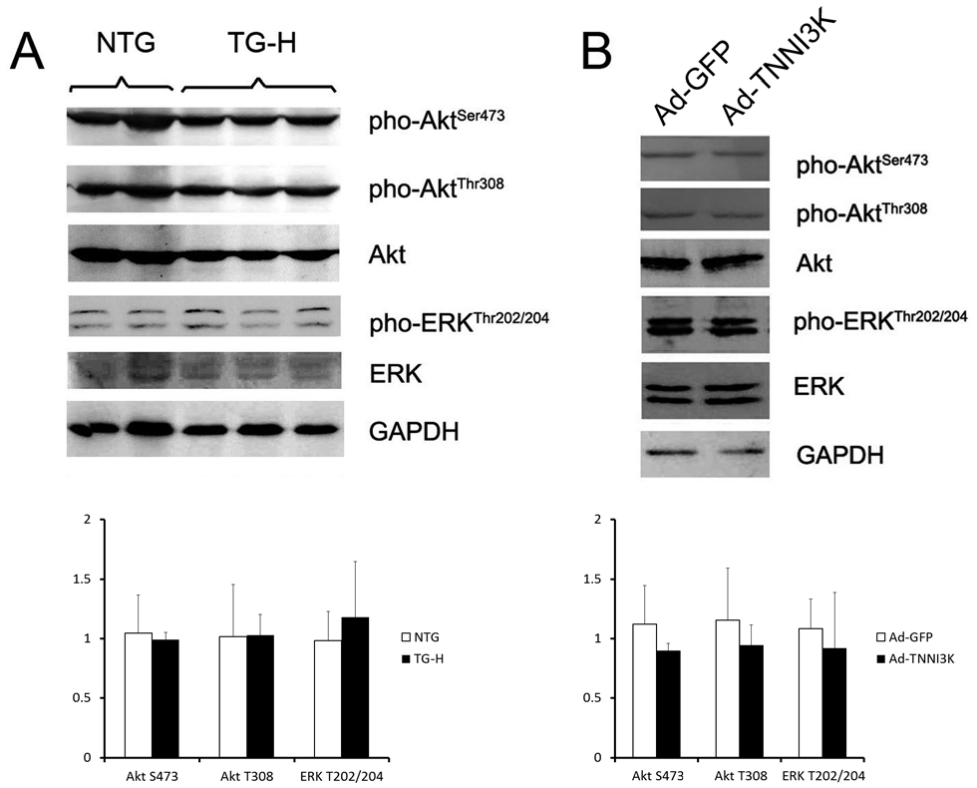
TNNI3K did not induce activation of Akt and ERK *in vivo* and *in vitro*. A: Western blot assessment for phosphorylation of Akt and ERK from transgenic hearts and nontransgenic control hearts at the age of 3 month. B: Cardiomyocytes were infected with Ad-TNNI3K and Ad-GFP for 48 hours and phosphorylation of Akt and ERK were analyzed by western blot. No significant phosphorylation increase was detected for the two effectors *in vivo* and *in vitro*. Each data point is shown as mean±SD.

Akt and ERK were among the best characterized signaling cascades that induce cardiac hypertrophy. In the left ventricular of TG-H mice at the age of 3 months, however, no significant difference was identified in the amount of total or phosphorylated Akt and ERK (Figure 5A). Consistent with the *in vivo* results, overexpression of TNNI3K in cardiomyocyte with a recombinant adenovirus does not induced activated of Akt or ERK (Figure 5B). Therefore, TNNI3K might not be involved in these two signal pathway.

### 3.5. TNNI3K interacts with cTnI and induced cTnI phosphorylation at Ser22/Ser23

**Figure 6:**
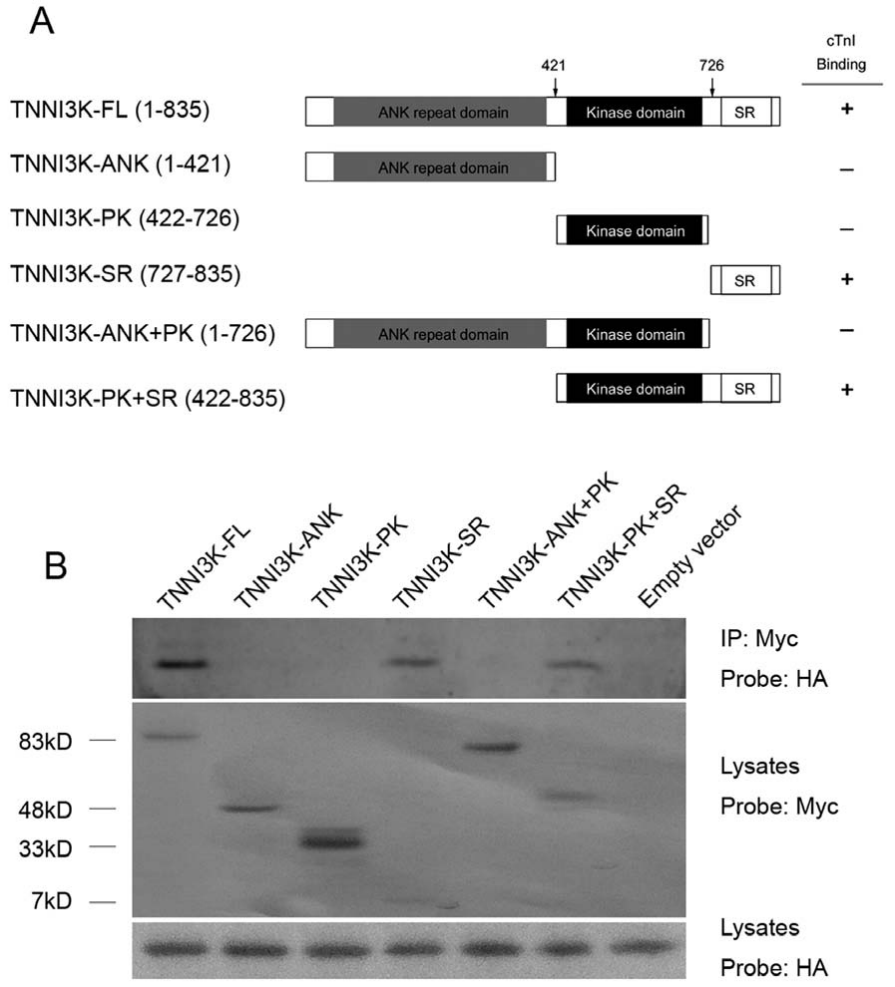
cTnI interacts with the serine-rich domain in the carboxyl terminus of TNNI3K. (A) Schematic representation of cTnI TNNI3K binding results. The selected domains on TNNI3K were indicated by labeled boxes. FL: full length, ANK: ANK repeat domain, PK: protein kinase domain, SR: serine rich domain. (B) H9C2 cells were transiently transfected with plasmids expressing full-length HA-tagged cTnI and the full-length Myc-tagged TNNI3K or the indicated truncations of Myc-tagged TNNI3K. TNNI3K was immunoprecipitated (IP) with anti-Myc antibodies and the presence of cTnI in the immunoprecipitate was assessed by immunoblotting with anti-HA antibodies. The expression of myc-TNNI3K from transfected constructs in the lysates is shown on the middle panel. The abundant of HA-cTnI in the lysates probed with anti-HA was used as an input.

To gain insight into the proteins those physiologically interact with TNNI3K in the heart, we performed the yeast two-hybrid study using full-length TNNI3K as bait. Screening of human heart cDNA library resulted in the identification of thirteen TNNI3K-interacting factors, one of which was cTnI. Previous data suggest there is a physical interaction between TNNI3K and cTnI[10]. To map the region of TNNI3K that bind cTnI, amino- and carboxyl-terminal truncations of TNNI3K were co-expressed with full-length cTnI, and the TNNI3K protein was immunoprecipitated. As expected, the full-length TNNI3K efficiently immunoprecipitated cTnI (Figure 6B, lane 1). cTnI also binds the truncations of TNNI3K lacking the amino-terminal ANK-repeat domain and/or the central protein kinase domain. However, once the carboxyl-terminal serine-rich domain was truncated, all detectable cTnI binding was lost (Figure 6B). These results indicate that TNNI3K binds to cTnI in its carboxyl-terminal region bound by amino acids 727 and 835, outside the protein kinase domain.

**Figure 7:**
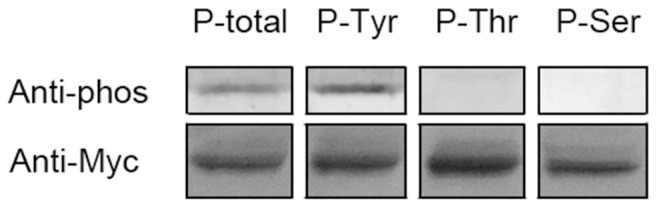
Detection of specificity of phosphoproteins in TNNI3K. Myc-tagged TNNI3K was overexpressed in H9C2 cells. TNNI3K proteins were immunoprecipitated with anti-myc antibody and Western blotted with anti-phosphoamino acid antibody (P-total), anti-phosphotyrosine antibody (P-Tyr), anti-phosphothreonine antibody (P-Thr), anti-phosphoserine antibody (P-Ser) (top panel) or anti-myc (bottom panel).

TNNI3K is a functional kinase. To identify the specificity of phosphoamino-acid, TNNI3K was overexpressed in H9C2 cells, immunoprecipitated, and immunoblotted with anti-phosphoamino acid antibodies. As shown in Figure 7, only phosphotyrosine, but no phosphoserine or phosphothreonine was detectable in TNNI3K, suggesting it is a protein-tyrosine kinase.

**Figure 8:**
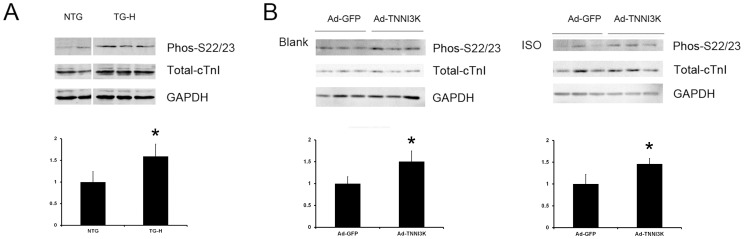
TNNI3K induced cTnI phosphorylation at Ser22/Ser23 *in vivo* and *in vitro*. (A), Heart lysates of TG-H and NTG mice were immunoblotted with antibodies against total and phosphorylated cTnI. TNNI3K induced cardiac troponin I phosphorylation at Ser22/Ser23 *in vivo*. (B), TNNI3K-induced cTnI phosphorylation at Ser22/Ser23 in cardiomyocytes on the basal and isoproterenol-stimulated level. Immunoblots are representative of 3 independent experiments. *: P<0.05 vs control group.

As TNNI3K is a functional kinase and it directly interacts of cTnI, we then performed immunoblot analysis to examine the effect of TNNI3K on cTnI phosphorylation. In the TNNI3K transgenic heart, the overexpression of TNNI3K was accompanied by increased cardiac troponin I phosphorylation at Ser22/Ser23 (Figure 8A). In cultured cardiomyocytes, infection with Ad-TNNI3K significantly induced cTnI phosphorylation at Ser22/Ser23 on the basal and isoproterenol-stimulated level, relative to cells infected with Ad-GFP (Figure 8B). Since the phosphorylation of troponin is a highly significant element in the control of cardiac contractility, these data suggested the role of TNNI3K in regulating cTnI phosphorylation and contractile function in the heart.

## Discussion

In this study, we found that TNNI3K is associated with cardiac remodeling induced by hemodynamic overload. Overexpression of human TNNI3K specifically in the heart of transgenic mouse lines caused long-standing concentric hypertrophy associated with enhanced cardiac pump function. Furthermore, TNNI3K directly interacts with cTnI and induced cTnI phosphorylation at Ser22/Ser23 *in vivo* and *in vitro*. These data suggest that TNNI3K promotes cardiac remodeling via regulating the phosphorylation of cTnI.

In consistent with previous studies *in vitro*,[14], [15] we found that overexpression TNNI3K could induce significantly enlargement of cardiomyocyte *in vivo*. Moreover, the TNNI3K transgene resulted in a concentric hypertrophy that is associated with enhanced cardiac function at the age of 3 months. There was no sign of pathological phenotype such as interstitial fibrosis. More importantly, the concentric hypertrophy remained up to 12 months of age and the left ventricular function was still normal. Therefore, we believe that the TNNI3K overexpression leads to an adaptive cardiac hypertrophy rather than maladaptive hypertrophy.

Cardiac hypertrophy is associated with alternation of cardiac gene expression.[21], [22] For example, physiologic hypertrophy of the heart is generally associated with the induction of α-MHC expression; however, during pathologic hypertrophy, β-MHC is increased at the expense of α-MHC. In TNNI3K transgenic hearts, although β-MHC was mildly up-regulated, the expression of α-MHC was significantly increased. This result is in agreement with the previous report, showing that the adenovirus-mediated TNNI3K overexpression increases the content of α-MHC in cardiomyocytes,[14] Moreover, the expression of SERCA2a is also increased in transgenic heart. As α-MHC-to-β-MHC ratio and SERCA2a-to-phosholamban ratio are positively correlated with left ventricular contractility,[23], [24] these molecular changes provide an explanation for the enhanced cardiac function and hyperhemodynamic state of TNNI3K transgenic heart.

To study the function of TNNI3K, we used “gain-of-function” strategy to generate transgenic mice in the C57BL/6J strain. Among the three transgenic lines, only the highest-copy-number transgenic mice developed a significantly cardiac remodeling. We believe the most likely explanation for this phenotype differences between the three lines of mouse is the endogenous counterbalancing mechanism. Firstly, C57BL/6J strain shows robust expression of TNNI3K in the heart at baseline.[16] Secondly, we generated transgenic mice expressing a wild-type CARK rather than a continuous-active CARK mutant. Therefore, lower expression level of exogenous CARK might be well tolerated by an endogenous counterbalancing mechanism.

Our observations with mice expressing TNNI3K are different from the previous report that described an accelerated disease progression in TNNI3K transgenic mice in response to pressure overload and in Csq transgenic mice.[16] This apparent discrepancy may be explained by the difference in the level of activation of TNNI3K signaling in the two mouse models. In Wheeler's study, the expression of TNNI3K in high-copy-number is comparable with that of low-copy-number transgenic lines. In our study, however, the difference in expression level is more than 20 folds. In addition, Wheeler et. al. found no major phenotype in the transgenic at baseline, which is consistent with our findings with the two lines that expresses low and moderate levels of TNNI3K. Hence, it is possible that because of differences in levels of activity of TNNI3K, pathways are less activated in Wheeler's mice than in our mice.

Up to date, little is known about the downstream targets of TNNI3K that are involved in the regulation cardiac hypertrophy. Given the facts that the IGF-1-phosphoinositide 3-kinase (PI3K)-Akt pathway and MEK1-ERK1/2 pathway are mainly advocated to mediate physiological cardiac growth,[2], [25] and the physiological hypertrophy demonstrated by TNNI3K transgenic mice is reminiscent of phenotypes displayed in transgenic mice expressing a caPI3K mutant[26] and caMEK1 mutant,[27] we hypothesized that the functional role of TNNI3K may be accomplished through the two signaling pathways. However, the activities of Akt and ERK were not changed by TNNI3K overexpression either *in vivo* or *in vitro*, suggesting TNNI3K promotes cardiac hypertrophy through other signaling pathway.

Previous study reported that TNNI3K directly binds to cTnI.[10] In our study, this interaction was confirmed independently both in the two-hybrid screen and in *in vitro* binding experiments. As the unique isoform expressed in heart muscle, cTnI plays a key role in the modulation of cardiac myofilament response to protein phosphorylation. cTnI can be phosphorylated at multiple amino acid residues by various kinases.[28] The phosphorylation level of cTnI at different residues has been proved to be a highly significant element in the control of cardiac contractility and a potential point of vulnerability in the network of signals by which hypertrophy and failure evolve.[29], [30] Ser22/Ser23 locates in the cardiac-specific N terminus of cTnI. Phosphorylation at the Ser22/Ser23 sites alters the shape of the cTnI, resulting in accelerated relaxation and augmented contractility[31]. In the present study, we found that TNNI3K overexpression induced cTnI phosphorylation at Ser22/Ser23 *in vivo* and *in vitro*, suggesting that TNNI3K is a novel upstream regulator for cTnI phosphorylation. Moreove, the TNNI3K transgenic mice demonstrate a unique hypertrophic phenotype with enhanced cardiac function and hyperhemodynamic state, which is consistent with the lusitropic effect of cTnI phosphorylation on Ser22/Ser23. Therefore, TNNI3K may promote cardiac remodeling via regulating the phosphorylation of cTnI.

In conclusion, our study shows that upregulation of TNNI3K was characterized by long-standing concentric hypertrophy with enhancement of cardiac function. This prohypertrophic effect of TNNI3K was associated with the increased cTnI phosphorylation on Ser22/Ser23. As the phosphorylation state of Ser22 and/or Ser23 is significantly reduced in end-stage failing hearts[32], [33], future studies investigating whether TNNI3K could be a potential therapeutic target for heart failure should be pursued.

## Supporting Information

Figure S1 **Schematic M-mode echocardiographic tracings of TG-H and non-transgenic littermates at the age of 3 month.** LVEDD: LV end-diastolic diameter, LVPWD: LV posterior wall thickness in diastole, IVSD: interventricular septum thickness in diastole.
(TIF)

Table S1 **List of real-time PCR primers for rat and mouse.**
(DOC)
