# Supplementary figures and images for: TNNI3K, a Cardiac-Specific Kinase, Promotes Physiological Cardiac Hypertrophy in Transgenic Mice
Source: PLoS One. 2013 Mar 5;8(3):e58570. doi: 10.1371/journal.pone.0058570 (PMC3589374; doi:10.1371/journal.pone.0058570)

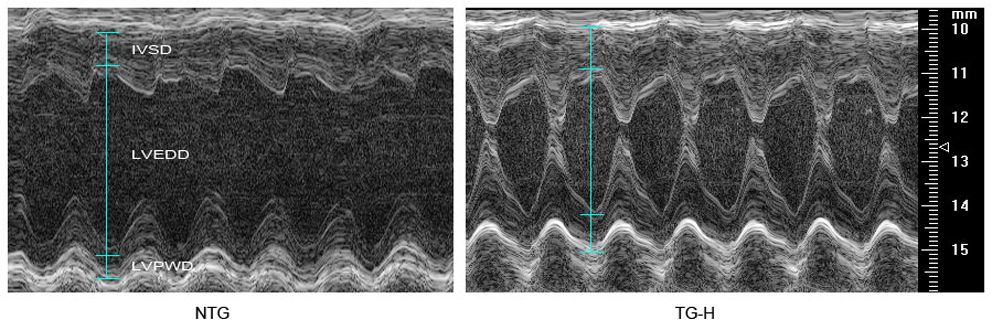

Supplement: Figure S1 — Schematic M-mode echocardiographic tracings of TG-H and non-transgenic littermates at the age of 3 month. LVEDD: LV end-diastolic diameter, LVPWD: LV posterior wall thickness in diastole, IVSD: interventricular septum thickness in diastole. (TIF) [file pone.0058570.s001.tif]
