# Supplementary material for: TNNI3K, a Cardiac-Specific Kinase, Promotes Physiological Cardiac Hypertrophy in Transgenic Mice
Source: PLoS One. 2013 Mar 5;8(3):e58570. doi: 10.1371/journal.pone.0058570 (PMC3589374; doi:10.1371/journal.pone.0058570)
Supplement: Table S1 — List of real-time PCR primers for rat and mouse. (DOC) [file pone.0058570.s002.doc]

Table S1 List of real-time PCR primers for rat and mouse.

| Species | Primer name | Sequence (5’-3’） | Product length(bp) |
| --- | --- | --- | --- |
| rat | rANP-RT-P1F | TCA GAG AGA TGG AGG TGC | 145 |
|  | rANP-RT-P1R | AAT CCT GTC AAT CCT ACC C |  |
|  | rTNNI3K-RT-P3F | CGC TTC CAT CTC CAA CTC TC | 128 |
|  | rTNNI3K-RT-P3R | TAG GTG TTG GCT CGG TAT CG |  |
|  | rGAPDH-RT-P6F | CTC TAC CCA CGG CAA GTT C | 151 |
|  | rGAPDH-RT-P6R | GCC AGT AGA CTC CAC GAC ATA |  |
|  |  |  |  |
| mouse | mGAPDH-P1F | GGC ATT GTG GAA GGG CTC | 210 |
|  | mGAPDH-P1R | GGG GGT AGG AAC ACG GAA G |  |
|  | mRPL7-P1F | GCT GCG GAT TGT GGA GCC ATA C | 170 |
|  | mRPL7-P1R | CCT CCA TGC AGA TGA TGC CAA AC |  |
|  | mBNP-P1F | CTT TAT CTG TCA CCG CTG GGA G | 167 |
|  | mBNP-P1R | TTT GGG TGT TCT TTT GTG AGG C |  |
|  | mANP-P2F | AGT GCG GTG TCC AAC ACA G | 123 |
|  | mANP-P2R | TGC TTC CTC AGT CTG CTC ACT C |  |
|  | mMyh6-P2F | TGC TGA GGG AAC AGT ATG AA | 120 |
|  | mMyh6-P2R | TCT GTA TGG CAT CCG TCT C |  |
|  | mMyh7-P2F | ACA GAG GAA GAC AGG AAG AAC C | 199 |
|  | mMyh7-P2R | GCT TGT TGA CCT GGG ACT C |  |
|  | mActa1-P2F | TTA TCG GTA TGG AGT CTG CG | 183 |
|  | mActa1-P2R | TCT TCA TGG TGC TGG GAG |  |
|  | mAtp2a2-P2F | CAG TTC ATC CGC TAC CTC | 118 |
|  | mAtp2a2-P2R | CCA GAT TGA CCC AGA GTA AC |  |
|  | mPLN-P1F | TCA CTC GCT CGG CTA TCA GGA GAG | 150 |
|  | mPLN-P1R | CGG CAG CTC TTC ACA GAA GCA TC |  |
